# Are cone beam CT image matching skills transferrable from planning CT to planning MRI for MR-only prostate radiotherapy?

**DOI:** 10.1259/bjr.20210146

**Published:** 2021-04-29

**Authors:** Rachel L Brooks, Hazel M McCallum, Rachel A Pearson, Karen Pilling, Jonathan Wyatt

**Affiliations:** 1 Northern Centre for Cancer Care, Newcastle upon Tyne Hospitals NHS Foundation Trust, Newcastle upon Tyne, UK; 2 Centre for Cancer, Newcastle University, Newcastle upon Tyne, UK

## Abstract

**Objectives:**

Treatment verification for MR-only planning has focused on fiducial marker matching, however, these are difficult to identify on MR. An alternative is using the MRI for soft-tissue matching with cone beam computed tomography images (MR-CBCT). However, therapeutic radiographers have limited experience of MRI. This study aimed to assess transferability of therapeutic radiographers CT-CBCT prostate image matching skills to MR-CBCT image matching.

**Methods:**

23 therapeutic radiographers with 3 months–5 years’ experience of online daily CT-CBCT soft-tissue matching prostate cancer patients participated. Each observer completed a baseline assessment of 10 CT-CBCT prostate soft-tissue image matches, followed by 10 MR-CBCT prostate soft-tissue image match assessment. A MRI anatomy training intervention was delivered and the 10 MR-CBCT prostate soft-tissue image match assessment was repeated. Limits of agreement were calculated as the disagreement of the observers with mean of all observers.

**Results:**

Limits of agreement at CT-CBCT baseline were 2.8 mm, 2.8 mm, 0.7 mm (vertical, longitudinal, lateral). MR-CBCT matches prior to training were 3.3 mm, 3.1 mm, 0.9 mm, and after training 2.6 mm, 2.4 mm, 1.1 mm (vertical, longitudinal, lateral). Results show similar limits of agreement across the assessments, and variation reduced following the training intervention.

**Conclusion:**

This suggests therapeutic radiographers’ prostate CBCT image matching skills are transferrable to a MRI planning scan, since MR-CBCT matching has comparable observer variation to CT-CBCT matching.

**Advances in knowledge:**

This is the first publication assessing interobserver MR-CBCT prostate soft tissue matching in an MR-only pathway.

## Introduction

The current gold-standard imaging for prostate radiotherapy planning is a CT scan.^1^ MRI provides better soft-tissue definition for target and organ at risk (OAR) delineation and is frequently integrated into the planning pathway and fused with the CT scan to exploit the soft-tissue benefits.^2^ Yet, the fusion of the CT and MRI introduces a systematic registration error, in addition to the inconsistencies in patient set up and organ positon, such as rectal and bladder filling.^1^ However, removing the CT scan from the planning pathway and using only the MRI scan to plan radiotherapy (MR-only) presents technical challenges as radiotherapy planning software requires electron density information acquired from the CT scan to calculate the dose distribution. This has been the focus of much research^2–4^ and commercially based solutions using a synthetic CT derived from the planning MRI scan have been evaluated.^5,6^

The principles of a MR-only planning pathway have been established in the literature.^1,7–9^ A challenge to the clinical implementation is daily verification of the patient’s position prior to treatment delivery, as this would conventionally be assessed by comparing the planning CT scan to the treatment cone beam computed tomography (CT-CBCT). Research has been conducted to evaluate the use of the synthetic CT for on treatment verification using fiducial markers,^10–12^ however fiducial markers can be difficult to accurately identify on MR.^13^ The use of fiducial markers requires an additional invasive insertion procedure and can have infection complications.^14^ In addition to the technical challenges of using fiducial markers in a MR-only pathway, 66% of UK radiotherapy centres use soft-tissue matching for on-treatment verification to visualise the prostate, seminal vesicle and OAR position.^15^ In which case, the synthetic CT provides poor quality images for soft-tissue matching as it does not accurately represent the patient anatomy, and is therefore only suitable for fiducial matching.^16^ Furthermore, relying on fiducial markers for treatment verification limits the MR-only pathway to the treatment of prostate cancer as fiducial markers are not routinely used for other cancers.

An alternative is using the MRI scan for on-treatment verification (MR-CBCT), however, therapeutic radiographers have limited experience of looking at MRI images and no experience of using them for online image matching.^17,18^ Despite that, therapeutic radiographers have extensive experience of image matching comparing a CT planning scan to a CBCT, and this recognition of anatomical structures could be a transferrable skill. This study aimed to evaluate the transferability of therapeutic radiographer image matching skills from CT-CBCT to MR-CBCT. The impact of focussed MR training will also be investigated.

## Methods and materials

### Observers

23 therapeutic radiographers with 3 months–5 years’ experience of CT-CBCT online daily soft-tissue matching prostate cancer patients at the Northern Centre for Cancer Care (NCCC), Newcastle upon Tyne, UK, participated in the repeated measures study. The therapeutic radiographers had local prostate soft-tissue image matching competency with no prior experience of MRI acquisition or MRI image matching.

### Image data

Two cohorts of data were used in three image matching assessments. Cohort 1 data were the CBCT acquired at the first treatment fraction for a random 10 consecutive patients who received a conventional CT based pathway. Cohort 1 data were used for the CT-CBCT image matching assessment 1. Cohort 2 data were the first fraction CBCT of the first 10 clinical MR-only patients and was used for the MR-CBCT image matching assessment 2 and assessment 3. Patients with a hip prosthesis were excluded from all cohorts, and patients larger than the MR field of view (FOV) were excluded from the MR-only cohort. The patients were consented for their data to be used for research, training and audit purposes as part of the consent for radiotherapy treatment. All patient data were anonymised prior to inclusion in this retrospective review.

For cohort 1 data, planning CT (Sensation Open, Siemens, Erlangen, Germany) scans were acquired on a flat couch top with standard immobilisation of head rest, knee blocks and foot stocks. Patients followed local bowel and bladder preparation before the scan and each treatment. Patients used a microenema 1 h before each procedure and emptied their bladder and drank 400 ml of water 30 min before each procedure. CT images were acquired with a tube voltage 120 kVp and 1.1 × 1.1 x 3 mm^3^ voxel size. FOV 550 mm, scan range from sacroiliac joint to 50 mm below inferior symphysis pubis.

For cohort 2 data, planning MR (1.5T Magnetom Espree, Siemens, Erlangen, Germany) images were acquired on a flat couch top with identical immobilisation and bowel and bladder preparation as cohort 1. MR images were acquired with a flexible 6-channel Body Matrix coil suspended on a coil bridge and the 24-channel Spine Matrix coil contained in the scanner couch. Images were a *T*_2_ weighted SPACE (Sampling Perfection with Application optimised Contrasts using different flip ad Evolution) sequence. This is a 3D turbo spin echo sequence with 450 × 450 mm^2^ FOV, scan range 180 mm, parameters are further described in Wyatt (2019).^16^

All patients were treated with 60 Gy in 20 fractions in a single VMAT arc on a Varian Truebeam STx (v. 2.7 MR3, Varian Medical Systems, Palo Alto, CA), following the same bowel and bladder preparation as their planning scans. All patients received daily kilovoltage CBCT, acquired with full arc, 465 × 465 mm^2^ FOV, scan range 120 mm, tube voltage 125 kVp, 900 mAs and voxel size of 0.9 × 0.9 x 2 mm^3^.

### Image matching skills assessment

Image matches were all completed in Aria (v. 13.7, Varian Medical Systems, Palo Alto, CA) using a non-clinical database. This simulated the clinical setting, yet facilitated the anonymisation of data and enabled the CBCT acquisition position to be set at 0 to ensure observers were not biased by previous matches. The auto-match within Aria is a rigid registration algorithm using mutual-information. This meant it could register images from different modalities and the same algorithm and settings were used for MR-CBCT and CT-CBCT registrations.

**Figure 1.**
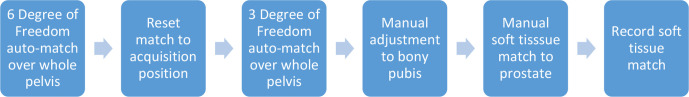
Diagram to show image matching process

The image matching process used in each assessment emulated the clinical process (Figure 1). It commenced with a 6 degree of freedom (6DoF) auto-match with the region of interest encompassing the whole pelvis to assess patient set-up for rotations. The 6DoF match was reset and a translations only automatch was carried out. This was followed by a manual adjustment to bony anatomy focussed on pubis due to its proximity to the prostate target. Finally, a manual soft-tissue match using the prostate outline to guide the match to the prostate and seminal vesicles, with consideration made to OAR due to bladder and rectal filling. The observer then recorded the vertical, longitudinal and lateral directional corrections they applied to complete the image match. Rotations were not recorded as these would not be applied in the clinical setting.

Assessment 1 required each observer to complete 10 CT-CBCT prostate soft-tissue matches offline. This was used as a baseline for interuser variability of current clinical practice. Assessment 2 required each observer to complete 10 MR-CBCT prostate soft-tissue matches without any MR training or experience. This was to assess image matching skills on a MRI planning scan. After completion of assessment 2 and prior to assessment 3, a 20-minute training intervention was delivered by one of the authors. The training intervention focused on MRI male pelvic anatomy, a comparison of structures visible on CT and MRI using the same MRI sequences to be used in the clinical setting and an example MR-CBCT image match. The content of the training intervention was compiled with multidisciplinary input from consultant clinical oncologists, physicists and MRI radiographers all with extensive MRI experience and delivered by a therapeutic clinical specialist radiographer with extensive image matching and MRI anatomy experience. Assessment 3 required each observer to repeat the 10 MR-CBCT prostate soft-tissue matches, blinded to the fact the images were the same as the images were re-labelled and reordered. Using the same images in assessment 2 and assessment 3 enabled a direct comparison and evaluation of the training intervention, blinding meant observers were not influenced by the image match decisions made in assessment 2. Additionally, to minimise learning from assessment 2, assessment 3 was completed at least 3 weeks after the completion of assessment 2. Assessment 3 was completed within five working days of the training intervention to ensure the training was being assessed in assessment 3.

### Statistical analysis

Statistical analysis was completed in Microsoft Excel. Interobserver error was calculated as mean standard deviation of all users of each image as used by McNair (2015).^19^ Interobserver variability was assessed using Jones (2011)^20^ Bland–Altman extension for multiple observers. Limits of agreement were calculated using Jones (2011)^20^ methodology and assess the disagreement of the observers with mean of all observers.^20^

## Results

**Figure 2.**
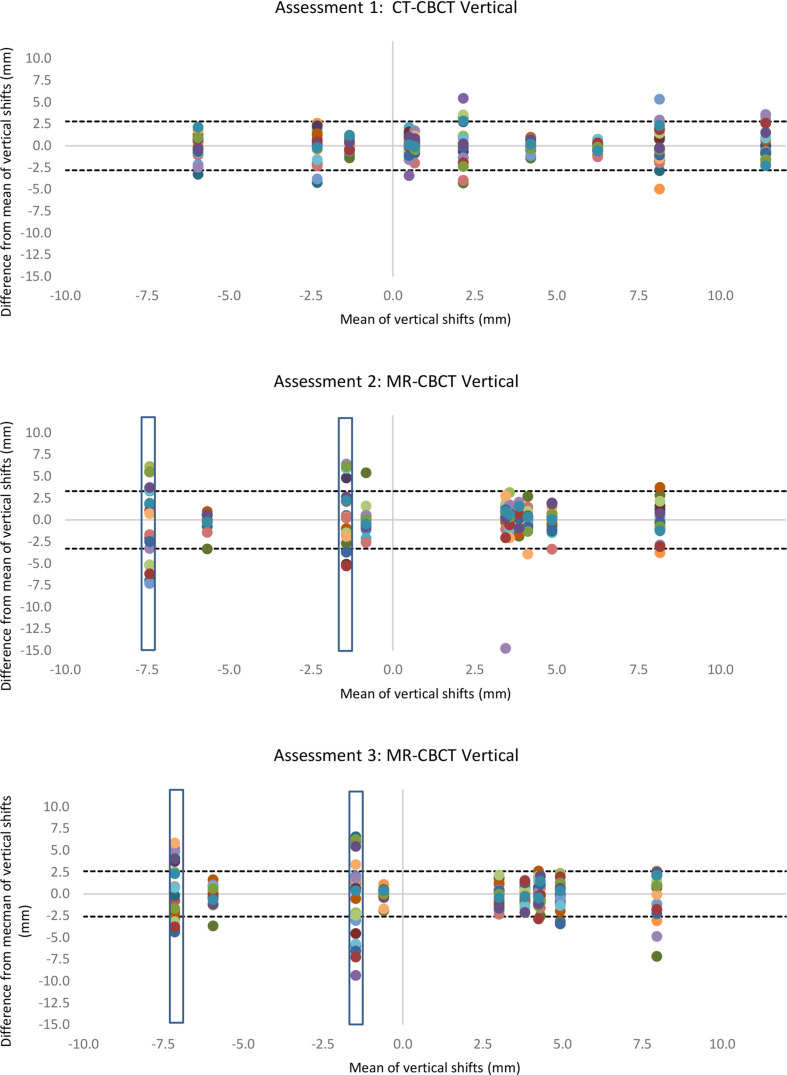
Modified Bland–Altman plots show vertical shifts of each radiographer image match compared to the mean of all radiographers (each radiographer is represented as a different colour) in three different assessments. The dotted lines show the limits of agreement with anomalies excluded. Blue box shows excluded data. CBCT, cone beam computed tomography.

**Figure 3.**
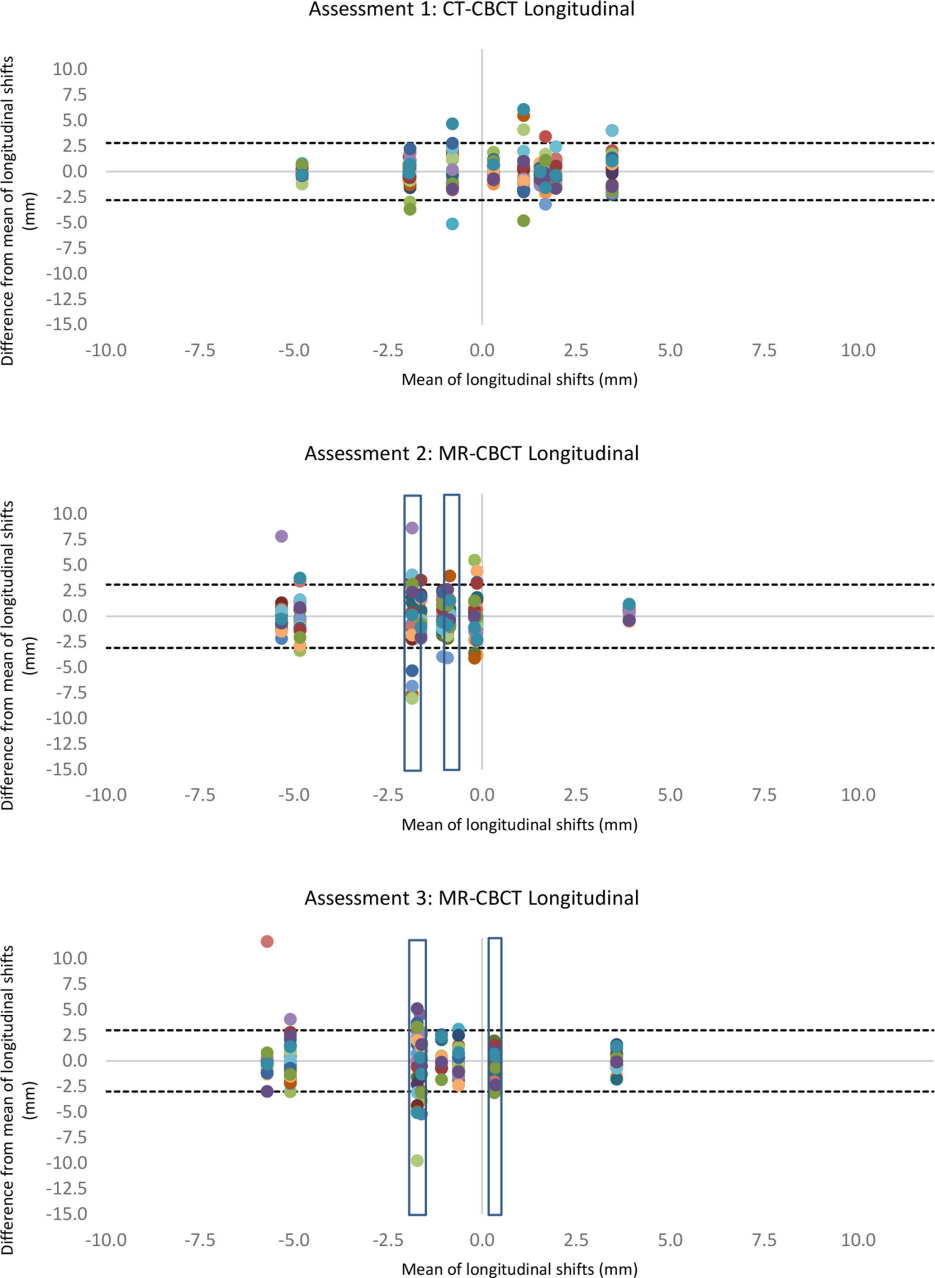
Modified Bland–Altman plots show longitudinal shifts of each radiographer image match compared to the mean of all radiographers (each radiographer is represented as a different colour) in three different assessments. The dotted lines show the limits of agreement with anomalies excluded. Blue box shows excluded data. CBCT, cone beam computed tomography.

**Figure 4.**
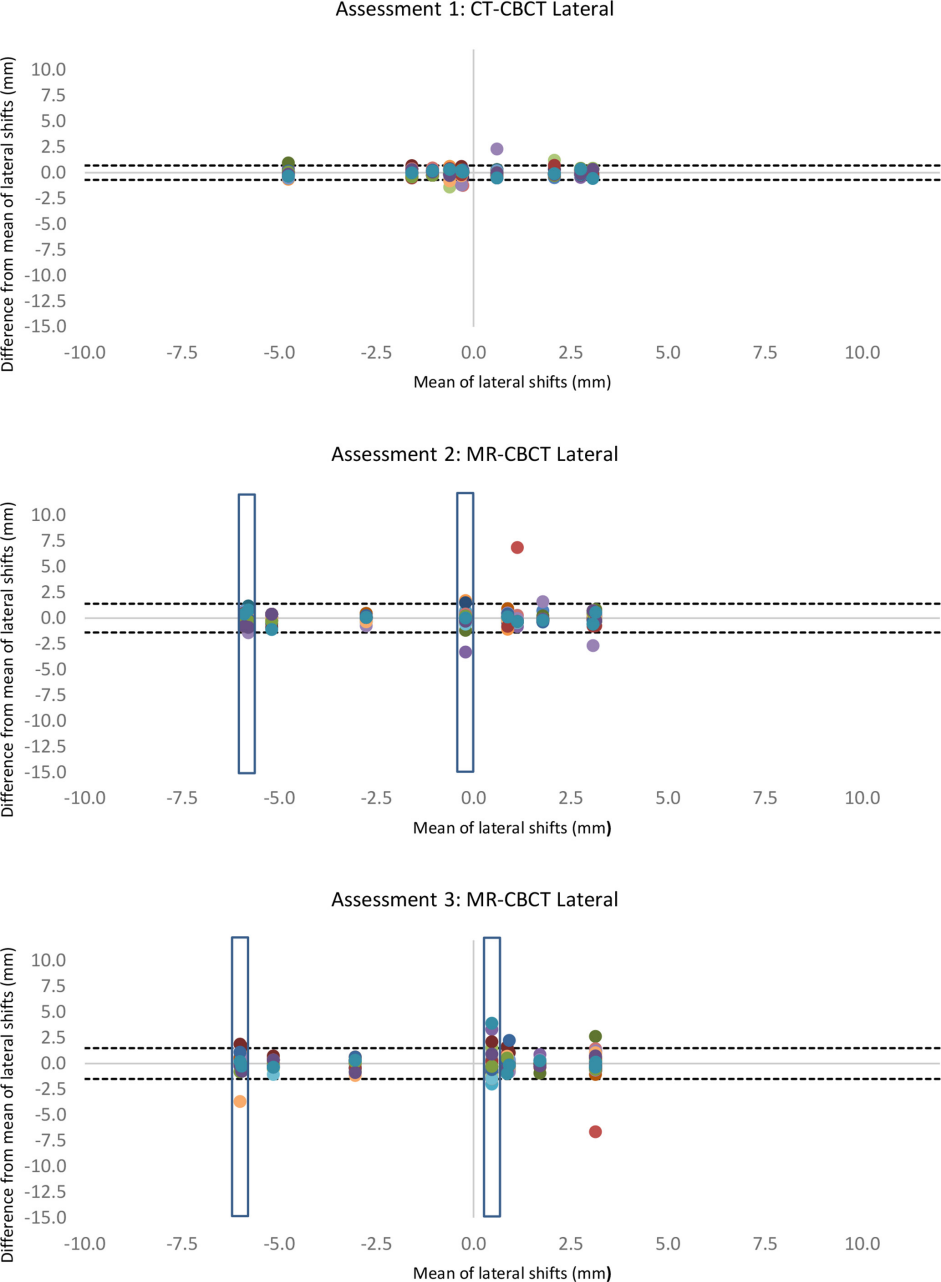
Modified Bland–Altman plots show lateral shifts of each radiographer image match compared to the mean of all radiographers (each radiographer is represented as a different colour) in three different assessments. The dotted lines show the limits of agreement with anomalies excluded. Blue box shows excluded data. CBCT, cone beam computed tomography.

**Figure 5.**
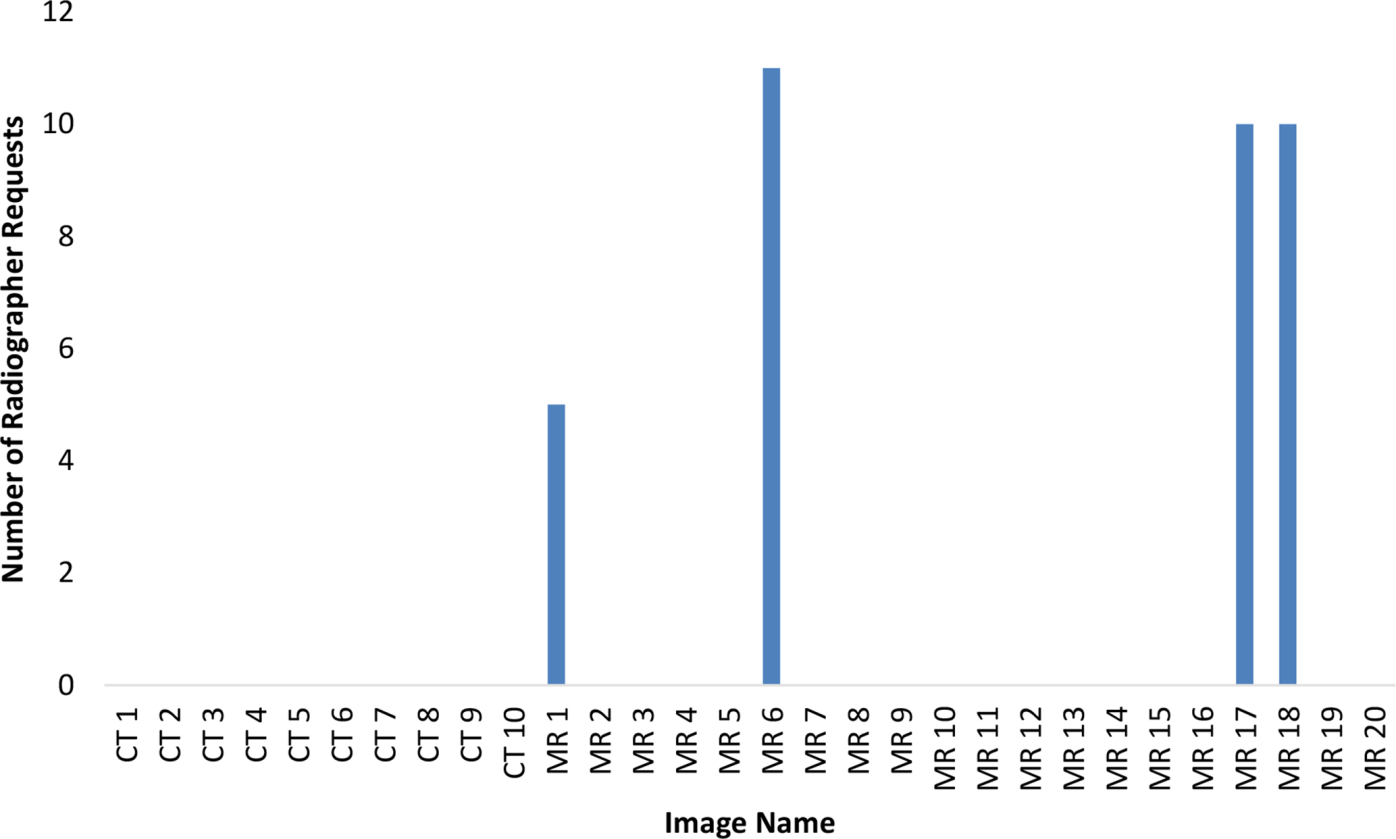
Radiographer rescan requests due to significant bladder or rectal differences on CBCT compared with planning scan. Patient MR 1 was the same image as patient MR 17, and patient MR 6 was the same image as patient MR 18. CBCT, cone beam computed tomography

Table 1 shows the interobserver error across all observers in the vertical, longitudinal and lateral directions of the image match. Bland–Altman plots demonstrated anomalies within the data collection (Figures 2–4). The variation was particularly evident in the vertical direction (Figure 2) and in one image match in the longitudinal direction (Figure 3). Additionally, annotations made by the observers highlighted that two CBCTs were difficult to match due to differences in patient bladder and rectal filling between the planning scan and the treatment CBCT. This was substantially more than the usual variation of bladder and rectal filling, however, not unique to MR-CBCT image matching. A number of observers stated that if this had been a clinical situation, they would have asked the patient to repeat their bladder and rectal preparation and rescan the patient as they did not deem the CBCT an appropriate treatment position, these rescan requests can be seen in Figure 5. These two CBCT images were excluded in the subsequent data analysis. Since the same images were used in assessment 2 and assessment 3 with relabelling, the images excluded were labelled; MR1, MR6, MR17, MR18. Table 1 shows excluding the anomalies has reduced the inter observer error, standard deviation and limits of agreement. There were no such apparent image anomalies in the baseline assessment 1 CBCT.

**Table 1.** Interobserver error of all observers for each assessment, bold italic shows data with two anomalous patients excluded

	Assessment 1 CT-CBCT		Assessment 2 MR-CBCT		Assessment 3 MR-CBCT	
(mm)	Interobserver error (±standard deviation)	Limits of agreement	Interobserver error (±standard deviation)	Limits of agreement	Interobserver error (±standard deviation)	Limits of agreement
							Vertical	1.3 (±0.7)	2.8	2.0 (±1.3)	4.6	1.9 (±1.3)	4.4
			***1.5 (±0.8)***	***3.3***	***1.3 (±0.5)***	***2.6***
Longitudinal	1.3 (±0.7)	2.8	1.8 (±0.9)	3.8	1.5 (±0.9)	3.5
			***1.5 (±0.5)***	***3.1***	***1.2 (±0.4)***	***2.4***
Lateral	0.3 (±0.1)	0.7	0.5 (±0.2)	1.1	0.7 (±0.3)	1.5
			***0.4 (±0.2)***	***0.9***	***0.6 (±0.2)***	***1.1***

The data with excluded anomalies demonstrate that interobserver error, standard deviation and limits of agreement are similar across the assessments. There is a small increase in interobserver error and limits of agreement in assessment 2, when observers were looking at MR-CBCT without MR training. Following the training intervention interobserver error, and limits of agreement in assessment 3 were reduced to values comparable to baseline assessment 1.

Consistent with the interobserver error, standard deviation and limits of agreement, Bland–Altman plots show the least variation in the lateral direction, with greater variation in the longitudinal and vertical directions.

## Discussion

This study assessed therapeutic radiographer image matching skills for a MR-only radiotherapy pathway. The limits of agreement presented in Table 1 and shown in Figures 2–4 show similar agreement between MR-CBCT image matching following training as the current standard practice CT-CBCT image matching. This demonstrates that image matching skills were transferrable from CT to MRI planning scan for MR-CBCT image matching.

Previous work by Wyatt (2019)^16^ demonstrated the accuracy of MR-CBCT prostate soft tissue matching in a small group of observers by comparing MR-CBCT matches directly with CT-CBCT. Wyatt (2019)^16^ found similar limits of agreement of 3.5 mm, 2.4 mm, 0.9 mm on MR-CBCT and 3.0 mm, 1.9 mm, 0.5 mm on CT-CBCT (vertical, longitudinal, lateral) to the results 2.6 mm, 2.4 mm, 1.1 mm on MR-CBCT and 2.8 mm, 2.8 mm, 0.7 mm on CT-CBCT in Table 1. To the best of the author’s knowledge, the interobserver variability and limits of agreement of prostate MR-CBCT soft-tissue matching has not been evaluated in other literature. McNair (2015)^19^ quantified interobserver error with different CBCT acquisition parameters fused with a planning CT and found interobserver error of at least 1.6 mm, 1.2 mm, 0.4 mm (vertical, longitudinal, lateral) which is similar (±0.3 mm) to results in Table 1 and within the standard deviations McNair^19^ reported.

Rogers (2020)^21^ evaluated cervix image matching with different imaging modalities, CBCT-CT, MR-CT and MR-MR. Their threshold for clinical acceptability was ±5 mm limits of agreement, and this was met in lateral and longitudinal directions, but they reported ±5.8 mm CBCT-CT, ±5.4 mm MR-CT and ±4.3 mm MR-MR in the vertical direction. Although they did not assess MR-CBCT prostate matching, all the limits of agreement in this study were less than 5 mm, and not clinically significant. The largest limit of agreement reported was 3.3 mm vertical in MR-CBCT assessment 2.

Fiducial markers are an option for image-guided radiotherapy, limits of agreement have been quantified by Deegan^22^ as 1.2 mm, 1.1 mm, 0.8 mm (vertical, longitudinal, lateral). Fiducial markers have a reduced interobserver error^14,22^ in comparison to the limits of agreement in Table 1 where variation is 2.6–3.3 mm, 2.4–3.1 mm and 0.7–1.1 mm (vertical longitudinal, lateral). However, fiducial markers are only feasible for use within the prostate gland, are an invasive insertion procedure^14^ and can be difficult to identify on MRI for a MR-only pathway.^13^ Additionally, fiducial markers do not allow therapeutic radiographers to assess planning target volume (PTV) coverage of the target or OAR position when used with planar kV images.^15^ The position of the seminal vesicles relative to the prostate can change depending on bladder and rectal filling. Soft-tissue matching enables the radiographer to adjust the match to ensure the seminal vesicles remain in the PTV if patient bladder or rectal filling is different to planning, or ask the patient to repeat bladder and bowel preparation to improve OAR position.

Variation reduced from assessment 2 to assessment 3 by 0.7 mm, 0.7 mm and an increase of 0.2 mm limits of agreement (vertical, longitudinal, lateral) after the training intervention which demonstrates that the training had an effect. However, training may not have been necessary in order to deliver safe radiotherapy since the difference between the interobserver error assessment 2 data and the assessment 1 data were only 0.2 mm, which was not clinically significant. Alternatively, it may have been the additional exposure to MR images that improved observers’ recognition of MR structures and subsequent decision-making. McNair (2015)^23^ describes a multifaceted training programme including lectures, anatomy training, self-directed learning, followed by an assessment. These components of training are encompassed by the training intervention and assessments making the self-directed learning and recognition of structures difficult to isolate from the formal training intervention. Nevertheless, training reduced the interobserver error and is recommended for confident and efficient decision-making when introducing a new technique in radiotherapy.^23^

There is a small increase (0.4 mm) in limits of agreement in the lateral direction in assessment 3 compared with assessment 1. Across the assessments, the least variability is within the lateral plane, this is likely due to the small amount of prostate organ motion left to right. Despite the increase in variation, it is the direction with the smallest value and is not clinically significant.

Consistent with previous research,^19^ there is more variation between users in the vertical and longitudinal translations, McNair (2015)^19^ suggested that the longitudinal discrepancies are due to difficulty in visualising the prostate on the planning CT images, but this would also apply to the CBCT images. There is a reduction in variation in the longitudinal direction in assessment 3 (2.4 mm) compared with assessment 2 (3.1 mm) and assessment 1 (2.8 mm) limits of agreement, interobserver error and standard deviation 1.2 mm (±0.4), 1.5 mm (±0.5), 1.3 mm (±0.7) (assessment 3, assessment 2, assessment 1). One explanation is the border between the prostate and bladder is easier to visualise on the MRI, meaning there is only one decision on the border (*i.e.* on the CBCT) rather than the CT and the CBCT. After the training intervention, the observers are more informed about their image match decision-making so that MR-CBCT matching is closer to “ground truth” which could account for the reduction in variation.

A limitation of the study is the assessment of one MRI sequence in one radiotherapy centre. A *T_2_* weighted SPACE sequence is evaluated in this study. Since MRI sequences are highly customisable and affect image quality, each radiotherapy centre would need to validate their own sequences for MR-only planning and image matching. Another limitation was the number of CBCTs reviewed. Due to the large sample of 23 observers each completing three image matching assessments, the number of CBCTs reviewed in each assessment was limited to 10 to reduce experimental attrition.

## Conclusion

This suggests that therapeutic radiographers’ prostate CBCT image matching skills are transferrable to a MRI planning scan, since it has been demonstrated that MR-CBCT matching has comparable observer variation to CT-CBCT matching. This would mean a MR-only pathway can be implemented using MRI reference data for online prostate soft-tissue matching without the need for fiducial markers.
